# Continuous positive airway pressure reduces the incidence of atrial fibrillation in patients with obstructive sleep apnea: A Meta-Analysis and Systematic Review

**DOI:** 10.51894/001c.34521

**Published:** 2022-09-06

**Authors:** Ziad Affas, Saif Affas, Kutiba Tabbaa

**Affiliations:** 1 Internal Medicine Henry Ford Macomb Hospital; 2 Internal Medicine Ascension Providence Hospital

**Keywords:** cpap, afib, atrial fibrillation, osa, obstructive sleep apnea, arrythmia

## Abstract

**INTRODUCTION:**

Obstructive sleep apnea (OSA) and atrial fibrillation (AF) are disorders that have increased in the United States during recent years. Earlier investigations have shown that underlying undiagnosed and unmanaged OSA plays a significant role in high rates and also as a trigger for newly diagnosed AF. Since the introduction of continuous positive airway pressure (CPAP) as a main therapy for OSA, more studies have evaluated the effect of CPAP on the development or recurrence of AF in OSA patients. However, sample sizes in a limited number of studies have been too small to conclude whether CPAP therapy has a significant effect on the development of AF in patients with OSA. The authors report results of their systematic review and meta-analysis summarizing what is currently known about the efficacy of CPAP for mitigating AF in patients with OSA.

**METHOD:**

The authors systematically reviewed the published reports on CPAP use and the incidence of AF from PubMed, Google Scholar, EMBASE, Web of Science, meeting abstracts, and Cochrane databases published between January 2015 and November 2021. Study data were extracted by two reviewers and a random-effects meta-analysis was performed using RevMan version 5.4.

**RESULTS:**

A total of 17 studies that met inclusion criteria were identified Studies included a total of 6,523 patients, 3,248 (49.8%) who used CPAP and 3,275 (50.2%) who did not use CPAP. In a random effects model, patients treated with CPAP showed a decrease in the incidence of AF (OR, 0.51; 95% CI; 0.27; 0.97, p = 0.04). High heterogeneity among the included studies was also observed (I2 = 97%).

**CONCLUSION:**

Our results add to the increasing evidence that CPAP therapy may reduce the incidence of development of AF in patients with OSA. Prospective future studies and clinical trials are needed to refine our understanding of the relationship between OSA and AF and how CPAP may reduce cardiovascular disease development.

## INTRODUCTION

Atrial fibrillation (AF) is a cardiac arrhythmia that is likely to develop in individuals with obstructive sleep apnea (OSA).^1^ AF is the most common arrythmia worldwide and it has a significant effect on morbidity and mortality.^2^ AF can be caused by variety of underlying conditions including OSA, hypertension, Chronic obstructive lung disease, hyperthyroidism, and valvular heart disease are other causes of AF.^3^

OSA involves intermittent upper airway obstruction during sleep, which results in poor sleep quality and nocturnal hypoxemia.^4^ This intermittent upper airway obstruction during sleep and nocturnal hypoxemia, if not treated, can develop into cardiac and metabolic syndrome comorbidities.^5^ OSA is also a significant risk factor for stroke, obesity, and diabetes.^6^

The global prevalence of OSA has increased drastically over the past decades, recent data from the United States and Europe suggesting that between 14% and 49% of middle-aged men have clinically significant OSA.^7^ A study by Gami et al. showed that approximately half of patients with OSA had AF, and that the relationship between OSA and AF was greater than the relationship between AF and other risk factors.^8^

The syndrome of OSA comprises obstructive apneas with recurrent awakenings, excessive tiredness in the daytime, and gasping.^9^ Women generally account for one-third of OSA patients, and a normal body mass index (BMI) is common in OSA patients, particularly in older adults and people from southeast Asia.^9^ Continuous positive airway pressure (CPAP) therapy is a form of positive airway pressure ventilation in which a constant level of pressure greater than atmospheric pressure is continuously supplied to the upper respiratory tract of OSA patients during sleep.^10^

The application of positive pressure may prevent upper airway collapse as it occurs in OSA.^9^ CPAP therapy is very effective for managing OSA, and it is regarded as an effective therapy for patients with AF.^11^ However, the acceptance of and compliance with CPAP use can be a limiting factor, since approximately 8% of patients stop using CPAP after their first night, and 50% of patients stop within the first year.

CPAP treatment has been demonstrated to reduce deaths and cardiovascular events and improves hypertension control.^12^ Of the several treatments available for OSA, CPAP is the most widely accepted and shown in randomized controlled studies and single case studies to be highly effective.^13–15^ Several studies have evaluated the influence of AF on CPAP efficacy, but sample sizes and the number of studies included in reviews have been too small to conclude whether CPAP therapy has a significant effect on the development of AF in patients with OSA.

### Purpose of Study

Due to these uncertainties, the authors investigated what is currently known about the efficacy of CPAP for preventing AF in patients with OSA. The aim of this systematic review and meta-analysis was to investigate the utility of CPAP therapy for preventing AF in patients with OSA based on the current state of research.

## METHODS

### Data sources and search string

A systematic review and meta-analysis in accordance with the preferred reporting items for systematic reviews and meta-analyses guidelines (PRISMA) was performed.^16^ PubMed, Embase, Google scholar, and Cochrane library were searched for the publication period from January 2005 until November 2021. The scope of the literature search was based on the population, intervention, control, and outcome (PICO) format.^17^ The population (P) of interest was patients with OSA aged 20 to 65 years; the intervention (I) was CPAP; the control (C) was non-CPAP use; the outcomes (O) were apnea hypopnea index (AHI) > 15/hour of sleep, occurrence of paroxysmal AF, and 14-day electrocardiogram (ECG) results.^18^

The authors’ literature search was performed using the following specific keys; [“CPAP” OR “CPAP User” OR “Continuous positive airway pressure”] AND [“a.fib” OR “Atrial Fibrillation” OR “Paroxysmal Atrial Fibrillation” OR “Fibrillation”] AND [“Sleep” OR “apnea’ OR”obstructive sleep apnea” OR “OSA”]. We also searched within the reference list to retrieve more articles, but due to the scarcity of articles on the topic of interest, we were unable to obtain any additional articles. The search strategy retrieved a total of 39 studies.

### Study selection

The exclusion criteria consisted of studies that reported the following outcomes and parameters: moderate-to-severe valvular heart diseases (regurgitation or stenosis); post-cardiac surgery outcomes; uncontrolled systemic hypertension or pulmonary hypertension; use of psychoactive or other drugs that could influence breathing patterns; and Epworth sleepiness scale > 10. Case reports, review articles, case series, and articles in a language other than English were excluded.

Full-text studies on the efficacy of CPAP for AF were included. The types of studies considered for the systematic review and meta-analysis were randomized controlled trials, systematic reviews, and meta-analyses. Two reviewers (2^nd^ author SA and 3^rd^ author KT) assessed each title and abstract for data and inclusion/exclusion criteria. Disputes were resolved by consensus.

### Data extraction and quality assessment

A predefined data collection sheet was used to gather the following information from the included studies: study design, last name of first author, year of publication, types of patients, criteria for OSA diagnosis, number of AF patients reported, and method of AF evaluation. The quality of the extracted studies was then evaluated using the Cochrane risk of bias guidelines.^19^

Data were extracted twice at different times by using the same search words to avoid any risk of bias. The defined range of risk of bias was low to high or unclear. The *Cochrane Handbook for Systematic Reviews of Interventions* was followed with a particular focus on random sequence generation, allocation concealment, blinding, outcome assessment, and selective reporting of selected studies, and the percentage of each measure was accessed through a visualization graph.

### Data evaluation and analysis

A random-effects meta-analysis was performed using RevMan version 5.4. by a statistician. The random-effects model was chosen because the true effect size underlying all studies was unknown, which would indicate the use of a fixed-effects meta-analysis; thus, the authors selected a more conservative approach.^20^ The chosen effect size calculated was the odds ratio (OR) with associated 95% confidence intervals (CI).

If only a baseline or final value was available, the mean change of the score was calculated by subtracting the final value from the baseline value. The I² test was used to assess the magnitude of heterogeneity. A 95% CI was used, and the possibility of publication bias was assessed using a funnel plot of the included studies and effect size against the standard error.^21^ A group of three statisticians lent their expertise to the data analysis for this study but declined any credit or acknowledgment for this article.

## RESULTS

### Included Studies

A total of 39 articles were retrieved from the database search. After removing duplicates, the remaining 30 references were assessed for further eligibility. During the process of reading through the abstracts and titles, nine (30.0%) papers were excluded, mainly because of insufficient outcome measures (myocardial infarction, OSA patient only) or insufficient information required for our study.

**Figure 1.**
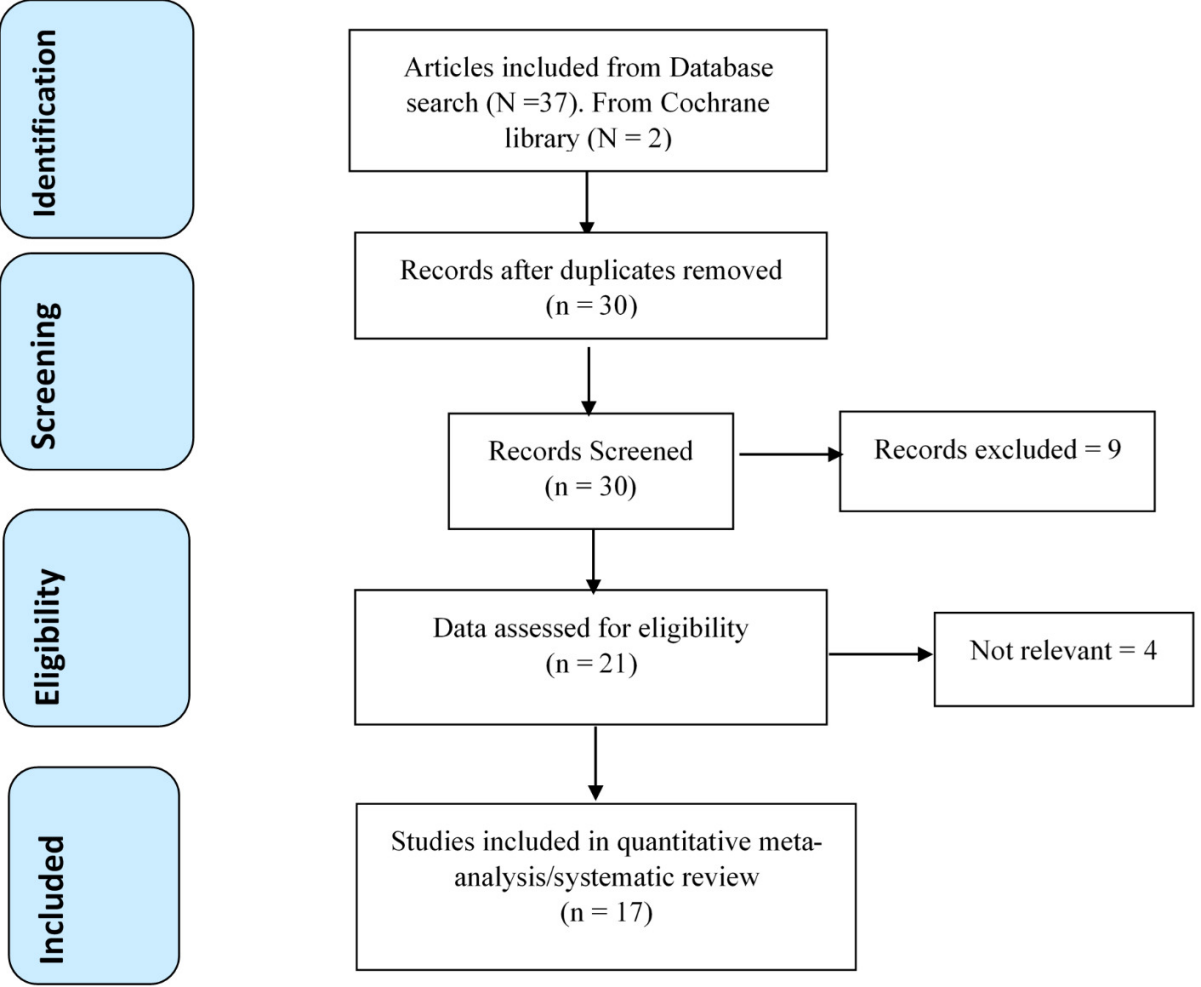
PRISMA Flowchart

Full text was examined in the remaining 21 articles, and of these, four (19.0%) were excluded for the following reasons: three articles reported CPAP for patients without OSA or were CPAP for AF trials without a control group, and one article was in a language other than English. The remaining 17 articles were considered for qualitative evaluation. (Figure 1)

### Study Characteristics

The 17 included studies were examined by first author ZA and third author KT, and the following data were extracted into a predefined excel sheet: last name of first author, study design, sample size, year of publication, type of patients, criteria for OSA diagnosis, method of administering CPAP, AF patients, AF therapy, method of AF evaluation, and the number of patients assigned to each group (intervention and control group). The results of the data extraction are shown in Table 1.

### Quality Assessment

**Figure 2.**
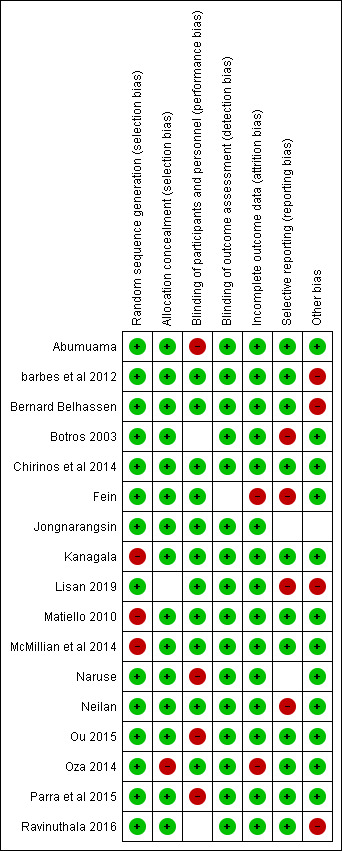
The summary of the reported quality assessment of the included studies The percentages of the grading are further summarized in Figure 3.

Of the 17 included studies,^22–38^ 14 were at “low risk of bias” under the random sequence generation, and three were at “high risk of bias.” For allocation concealment, 16 studies were of low risk, one of high risk. For blinding of participants and personnel, 12 were of low risk, one was of unclear risk, and four were of high risk. For blinding of outcome assessment, all the studies were of low risk. For incomplete outcome data, 15 were of low risk, two were of high risk, and zero were of unclear risk. For selective reporting bias, 11 studies were of low risk, four were of high risk, and two were of unclear risk. For other bias, 12 studies were of low risk, four were of high risk, and one was of unclear risk. (Figure 2)

**Table 1.** Characteristics of the included Studies

**First Author**	**Year**	**Design**	**Country**	**Type of Patients Included**	**Criteria for OSA Diagnosis**	**AF Patients Reported**	**Method of AF Evaluation**
Abumuamar ^22^	2018	PCS	Canada	OSA diagnosed by Polysomnography	Not Reported	Yes	EEG, snore, respiratory effort, pulse rate, and pulse waveform
Barbes ^23^	2012	RCT	USA	AF with apparently healthy patients	AHI >20+ESS≤10	Yes	Incident Hypertension of Mace
Bernard Belhassen ^24^	2013	PCS	USA	Polysomnography before AF ablation	AHI >15/h and >80% events had to be obstructive	Yes	12-lead ECG documentation
Botros ^25^	2003	RCT					
Chirinos ^26^	2014	RCT	USA (Pennsylvania)	AF with OSA	Not Reported	Not Reported	CRP level, insulin sensitivity, lipid levels, and blood pressure
Fein ^27^	2013	PCS	Japan	Polysomnography before AF ablation	AHI >15/h and >80% events had to be obstructive	Yes	12-lead ECG on clinic visits and transtelephonic monitoring
Jongnarangsin ^28^	2010	PCS	USA	Polysomnography before AF ablation	Not Reported	Yes	symptoms evaluation
Kanagala ^29^	2003	Observational Study	USA,	AF with OSA	AF/AFL referred for cardioversion	Yes	Clinical or ECG
Lisan ^30^	2019	Review	USA	CPAP and OSA	Moderate AHI>20	Not Reported	Not Reported
Matiello ^31^	2010	PCS	USA	Polysomnography (Patients with OSA)	Not Reported	Yes	Event monitor
McMillan ^32^	2014	RCT	USA	Comorbidity	Moderate AHI>15	Yes	Not Reported
Naruse ^33^	2013	PCS	JAPAN	Polysomnography 1 week after AF ablation	AHI >15/h and >50% events had to be obstructive	Yes	12-lead ECG documentation
Neilan ^34^	2013	PCS	USA	OSA diagnosed before PVI with polysomnography	AASM criteria	Yes	ECG or prolonged cardiac monitoring
Ou ^35^	2015	NRCS	USA	AF patients	Not Reported	Yes	ITT APPROACH
Oza ^36^	2014	PCS	USA	AF with polysomnography	AHI >15	Yes	Close Monitoring
Patel ^37^	2008	PCS	USA	polysomnography before PVI	Not Reported	Yes	Event monitor/Holter monitor
Ravinuthala ^38^	2016	PCS	USA	polysomnography	AHI of >5 and <15 was considered “Mild”. An AHI of 15 to<30 was considered “Moderate”. An AHI of 30 or more was considered “Severe”.	Yes	Event Monitor

Abbreviations: AASM, American Academy of Sleep Medicine; AF, atrial fibrillation; AFL, atrial flutter; AHI, apnea hypopnea index; CRP, C-reactive protein; ECG, electrocardiogram; EEG, electroencephalogram; OSA, obstructive sleep apnea; PCS, Prospective Cohort Study PVI, pulmonary vein isolation; RCT, randomized controlled trial; USA, United States of America; ITT, Intention to treat.

**Figure 3.**
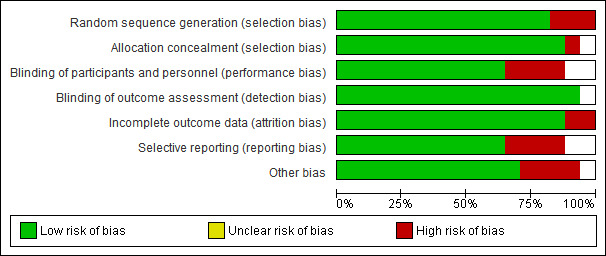
The percentage of the level of quality assessment of the included studies (Studies included in the meta-analyses for efficacy of CPAP for AF).

A total of 17 studies were included in the meta-analysis with a total number of 6,523 patients: 3,248 (49.8%) patients in the CPAP users group and 3,275 (50.2%) patients in the non-CPAP user group. The intervention group included CPAP users and the control group included non-CPAP users. The effect size was measured using the OR method and a random effect meta-analysis was carried out on the included studies.

### The efficacy of CPAP for AF in Patients with OSA

A random effect meta-analysis was performed to calculate an OR and 95% CI, since the effect size measure was used in the studies that were deemed fit for evaluating the efficacy of CPAP for AF in patients with OSA compared to the non-CPAP AF trials. The use of CPAP for AF trials showed a significant positive reduction in AF complications (OR = 0.51; 95% CI, 0.27-0.97; *p* = 0.04). The percentage of heterogeneity across studies (I^2^ = 97%) was high and statistically significant (*p* < 0.01), and the Z-score statistics were normally distributed (Z-score = 2.06).

**Table 2.**
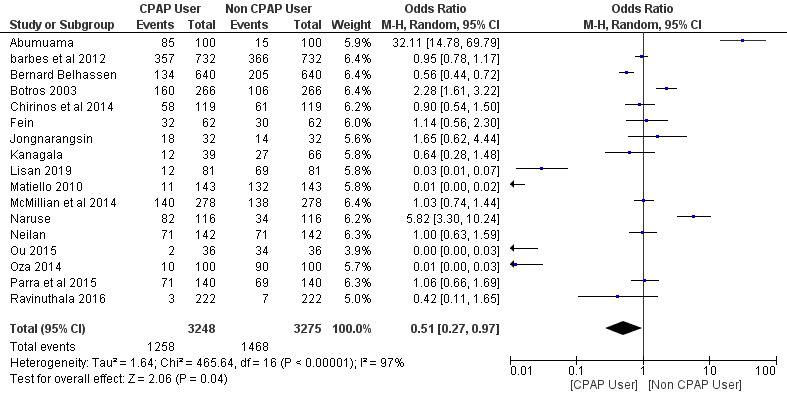
A forest plot of the comparison between CPAP users and non CPAP users

### Publication bias

**Figure 4.**
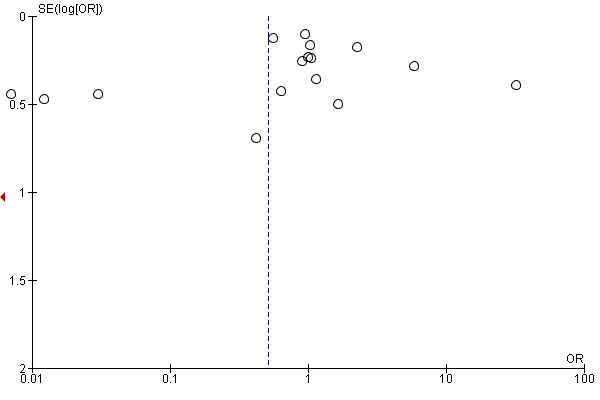
A funnel plot of the comparison

To assess publication bias across studies, visual inspection of a funnel plot was performed on all comparisons for the studies included in the meta-analyses. The funnel plot of studies included in the trials assessing efficacy of CPAP for AF demonstrated a symmetrical funnel with a few outliers, which indicates true heterogeneity rather than publication bias. (Figure 4)

## DISCUSSION

The results of our meta-analysis add to the literature summarizing the current literature indicating that CPAP therapy may reduce the likelihood of AF development in patients with OSA. Our results are similar and consistent with a previous meta-analysis by Qureshi et al., which found a significant effect of CPAP as a therapy for AF in patients with OSA.^39^ In that 2015 analysis, the authors examined eight studies and reported the rate of AF in 698 patients who used CPAP and 549 participants who did not use CPAP.^39^

By using the risk ratio (RR) as the effect size measure, they found that patients who used CPAP had an approximately 42% decreased risk of AF (RR, 0.58; 95% CI 0.47-0.70; *p* < 0.01), similar to our OR results indicating a benefit to CPAP use for reducing AF (OR, 0.51; 95% CI, 0.27-0.97; *p* = 0.04).

In our updated analysis, we were able to gather twice the number of studies, which collectively contained more than 3,000 patients in both the CPAP and non-CPAP using cohorts. Interestingly, we observed high and significant heterogeneity across all studies compared to the low heterogeneity of 30% that was seen by the Qureshi group.^39^ The authors hypothesize that this difference could be due to the current systematic review and meta-analysis having a larger number of studies and therefore also a larger number of patients included in aggregate.

Underlying physiological conditions, such as obesity, may feed into the complexity of how CPAP affects AF in people with OSA.^40^ This can be explained by increased upper airway collapsibility and local fat deposition leading to impaired neuromuscular control of the upper airway patency.^40^ Out of a total number of 3,542 patients, AF occurred in 114 of 2,626 (4%) patients who had OSA and in 19 of 916 (2%) patients without OSA, suggesting that OSA is a strong predictor of AF (hazard ratio, 2.18; 95% CI, 1.34-3.54; *p* < 0.01).^40^ Overall, that study suggested that obesity and the magnitude of nighttime oxygen desaturation (a consequence of OSA) may be independent risk factors for development of AF.

In addition, Two of the statisticians performed a multivariate regression analysis that did not reveal that CPAP use reduced incident AF in patients with OSA. However, the authors note that this finding may have been limited because the study did not account for CPAP use frequency, compliance, or effectiveness in treating apneas, and more importantly, because patients using CPAP often have more severe OSA, which may be a confounding factor.

In addition, inflammation, vascular endothelial dysfunction, increased sympathetic tone, and oxidative stress in patients with OSA may also contribute to development of AF.^41–43^ In addition a genetic link between OSA and risk of atrial fibrillation has also been posited, further suggesting that the connection between OSA and AF is very complex.^44^

To date, CPAP is considered the best therapy for patients with OSA. However, AF may recur in patients with OSA even after treatment with catheter ablation. Matiello et al.^31^ and Jongnarangsin et al.^28^ saw that patients with OSA had a higher recurrence of AF after catheter ablation, regardless of CPAP use.

Another study by Patel et al.^37^ showed that patients who were not treated with CPAP had a significantly higher early AF recurrence rate than those who had used CPAP, suggesting a benefit of CPAP therapy on preventing post-ablation AF recurrence. In 2013, Fein et al. reported that the atrial tachyarrhythmia-free survival rate was significantly higher in patients with OSA who were CPAP users than in CPAP non-users (71.9% v 36.7%; *p* = 0.01) and similar to that of patients without OSA (66.7%; *p* = 0.94).^27^

A separate meta-analysis by Shukla et al. observed a 42% relative risk reduction of AF recurrence with CPAP use in patients with OSA independent of medical or catheter ablation treatment approach, and they concluded that pulmonary vein isolation offered little benefit toward AF reduction in patients with OSA who were noncompliant with CPAP use.^39^ Finally, a 2020 analysis investigated the efficacy of CPAP in preventing multiple cardiovascular events, such as stroke, myocardial infarction, heart failure, unstable angina, and AF, in patients with OSA.^45^ They observed a relative risk of 0.94 (CI, 0.54-1.64) for CPAP prevention of AF.

Overall, treatments for AF, such as catheter ablation, may fail or succeed depending upon patients’ underlying conditions, such as OSA, and although current evidence strongly suggests that CPAP therapy may reduce post-treatment AF recurrence in people with OSA, the severity of OSA and the quality of AF treatment may also have an effect, and larger more detailed studies are needed. More prospective studies that address severity of OSA and individual cardiovascular pathologies, such as AF, are required to more rigorously explore whether CPAP therapy may have different effects on patients with various cardiac conditions and levels of OSA.

### Study Limitations

The first limitation was our lack of access to some of the articles needed for the research due to location and scarcity of topics. A second limitation was that subjects in the studies we included reported many outcomes, whereas our analysis was limited to patients with OSA. Third, our inability to extend our meta-analysis results to deeper analyses (e.g., meta-regression, sensitivity and specificity analysis) may also have been limiting.. Finally, the low number of included studies and the fact many of the studies analyzed (~86%) were not randomized controlled trials are also limiting. Therefore, the results of the current study should be interpreted with caution and should be utilized as hypothesis generating.

## CONCLUSIONS

Our meta-analysis results suggests that use of CPAP therapy may reduce development of AF in patients with OSA. Based on our findings, decreases in AF incidence from CPAP use may have extra-benefits of decreasing the need of using medications to control heart rate and anticoagulation, also reduce the risk of hospitalization, stroke, death, and the cost on the health system. More controlled studies will be needed to more rigorously examinee the apparent efficacy of CPAP for reducing AF.
